# Chloride Resistance of Portland Cement-Based Mortar Incorporating High Aluminate Cement and Calcium Carbonate

**DOI:** 10.3390/ma13020359

**Published:** 2020-01-12

**Authors:** Yunsu Lee, Seungmin Lim, Hanseung Lee

**Affiliations:** 1 Department of Architectural Engineering, Hanyang University, Ansan 15588, Korea; yslee23@hanyang.ac.kr; 2 Department of Architecture, Kangwon National University, Chuncheon 24341, Korea; smlim09@gmail.com

**Keywords:** chloride diffusion, chloride binding capacity, capillary pore volume, Portland cement, aluminate cement, calcium carbonate

## Abstract

Whether chloride resistance is highly influenced by chloride binding capacity remains unknown. In this study, the chloride resistance of Portland cement-based mortar incorporating aluminate cement and calcium carbonate was investigated considering the chloride binding capacity, pore structures and chloride diffusion coefficient from non-steady state chloride migration and natural chloride diffusion. The cement hydrates were investigated using X-ray diffraction and thermogravimetric analysis. The chloride binding capacity was evaluated based on the chloride adsorption from the solutions using the adsorption isotherm. The aluminate cement, as an available alumina source, can stimulate the formulation of layered double hydroxides, which in turn can increase the chloride binding capacity. The results of mercury intrusion porosimetry show that non-substituted (control) and substituted (only aluminate cement) specimens have capillary pore volume 8.9 vol % and 8.2 vol %, respectively. However, the specimen substituted with aluminate cement and calcium carbonate shows a higher capillary volume (12.9 vol %), which correlates with the chloride diffusion coefficient. Although the specimen substituted with calcium carbonate has a higher chloride binding capacity than the control, it does not necessarily affect the decrease in the chloride diffusion coefficient. The capillary pore volume can affect not only the chloride diffusion but also the chloride adsorption.

## 1. Introduction

Chloride does not directly affect the deterioration of concrete but the chloride attack on steel-reinforced concrete causes severe problems due to steel corrosion [1]. Thus, one of the major issues regarding the resistance of concrete to chloride ingress is improvement of the chloride-resistant ability of the binder, which acts as the passage of the fluid.

The approaches for the durability design of concrete in a high saline environment are based on factors such as the water-to-cement ratio, concrete strength and minimum values for unit cement content [2,3]. These are essential factors for the service life design of the concrete because the properties considered in most standards and codes for concrete durability [2,3] are related to the permeability of concrete and are influenced by the porosity, particularly the capillary pores. Thus, it is essential to reduce the capillary pore size or to increase the tortuosity of the cement matrix for the reduction of chloride transportation. For example, ground granulated blast-furnace slag, a representative supplementary cementitious material (SCM) for production of sustainable binders, is usually used to improve chloride resistance as it partially substitutes ordinary Portland cement (OPC) [4,5]. Not only can it refine the pore structure through the pozzolanic reaction but it can induce an increase in the chloride binding capacity, which also influences the chloride resistance of a cement matrix [6,7].

An increase in the chloride binding capacity can reduce the free chloride content at the equivalent total chloride [8,9]. This is because chloride exists in concrete as ions in the pore solution (free chloride) or as a bound state (bound chloride) in the form of chloride-bearing layered double hydroxides, such as aluminoferrite mono-substituted (AFm) phases and the adsorbate on the surface and/or interlayer of the hydrates, such as C-S-(A)-H [6,7,8]. In particular, it has been reported that chemical adsorption by the anion exchange mechanism in the layered structure has a higher chloride binding capacity than that of physical adsorption on the hydrates surface [10,11]. However, C-S-(A)-H can dominate the chloride binding behavior because the AFm phase content is quite limited in an OPC-based system. Falzone et al. [12] found the solution by taking advantage of the AFm phases. They investigated the chloride adsorption of the binder with high portions of AFm phases containing nitrate ions. Their research showed that the binder could be a functional coating material with a high chloride binding capacity as well as a corrosion inhibitor. However, the paste content per unit volume of mortar and the porosity in their study were not considered for the evaluation of the chloride resistance. Thus, it remains uncertain that the higher the AFm phase content, the higher the chloride resistance.

The pore characteristics should in no way be ignored, as well as the chloride binding capacity, when considering the chloride resistance. As various materials have different hydration kinetics and chemical compositions, they can influence both the cement hydrates [13,14] and pore structure [14,15] in an OPC-based system. Zajac et al. [16] reported that the pore structure of binders comprising similar chemical compositions could be different due to the hydration kinetics, because coarse pores can appear with an increase in the proportion of (semi-) crystals during initial hydration. Although not all AFm phases are crystalline (these phases are often poorly crystalline), this result shows that such a production of (semi-) crystalline AFm phases may induce coarse pores. Therefore, the different hydration kinetics can cause an increase in the capillary pores, which may in turn increase the chloride diffusion coefficient regardless of the chloride binding capacity.

In this regard, we questioned whether the high chloride binding capacity would have a decisive role on the chloride diffusion behavior in a cementitious material. This study utilizes high alumina cement (HAC) and calcium carbonate (CaCO_3_) to control the type and the amount of AFm phases in OPC-based system. The HAC is used as the alumina source to increase the available alumina in the OPC system. The available alumina can stimulate the formation of AFm phases (CAH_10_ and/or C_2_AH_8_), which can be converted to the various types of layered double hydroxide structure [17,18]. For this reason, the partial replacement of OPC with materials containing high Al_2_O_3_ content can increase the chloride binding capacity of an OPC-based system [9,17,18,19,20,21]. CaCO_3_ is additionally added to the binders to change the hydration kinetics and the type of AFm phases. Although CaCO_3_ has little reactivity, it can accelerate cement setting by inducing the formulation of carbonate-bearing alumina phases [22,23], by acting as a nucleation seed [24,25].

In this work, we report our findings on the chloride diffusion properties of OPC-based mortar blended with HAC and CaCO_3_ considering the pore characteristics and chloride binding capacity. The chloride diffusion coefficient of the mortar is discussed with regard to the chloride binding capacity and pore characteristics.

## 2. Materials and Methods

### 2.1. Materials

Type I OPC (Ssangyong cement, Korea), HAC with Al_2_O_3_ of ~70 wt % (UAC 70N, Union, Seoul, Korea) and extra pure grade CaCO_3_ (Daejung chemicals and metals, Siheung, Korea) were used in this study. Table 1 shows the oxide composition and the loss on ignition of the OPC, HAC and CaCO_3_. The oxide composition was analyzed by X-ray fluorescence spectroscopy (Axios, PANalytical, Almelo, The Netherlands) and the loss of ignition was measured by thermogravimetric analysis (TGA; DTG-60, Shimadzu, Japan). The TGA was conducted with a N_2_ purge of 20 mL/min at the heating rate of 20 °C/min, from 40 to 1000 °C and the hold time at 990 ± 10 °C was 15 min. The HAC was comprised of calcium mono-aluminate (CaO·Al_2_O_3_, CA) in primary phase and calcium di-aluminate (CaO·2Al_2_O_3_, CA_2_) in secondary phase. The chloride solutions were prepared with NaCl (Daejung chemicals and metals, Siheung, Korea).

### 2.2. Specimen Preparation

Table 2 shows the mix proportions of the specimens. Three types of binder, P (OPC 100%), A (OPC 85% + HAC 15%) and C (OPC 75% + HAC 15% + CaCO_3_ 10%), were used in this study. The cement paste and mortar were prepared using a Hobart mixer according to ASTM C305 [26].

The paste specimens with a water-to-binder (w/b) ratio of 0.4 were cured in a sealed ø30-mm conical tube at 20 ± 1 °C. The mortar specimens with a w/b ratio of 0.5 and a sand-to-binder ratio of 3 were casted in a 50 × 50 × 50 mm^3^ cubic (for compressive strength) or a ø100 × 200 mm^3^ cylindrical (for chloride migration or natural diffusion test) mold. The de-molding of all mortar specimens was conducted after 1 day, then, the specimens were cured in a water bath at 20 ± 1 °C. The compressive strength of the mortar specimens PM, AM and CM at 3 days were 20.3 (standard deviation (*σ*) = 1.8), 19.3 (*σ* = 1.0) and 18.2 (*σ* = 1.6) MPa, respectively and at 56 days were 34.1 (*σ* = 0.9), 24.0 (*σ* = 1.8) and 24.8 (*σ* = 1.3) MPa, respectively. The compressive strength test was conducted using a universal testing machine (DEC-30TC, Dawha, Korea) at a loading rate of 0.3 MPa/s and each specimen type was measured three times.

### 2.3. Heat of Hydration

The heat release rate and accumulated hydration heat of the paste were measured using an isothermal calorimeter (TAM-Air, TA Instruments, New Castle, DE, USA) for 72 h at 20 °C. An amount of 3.57 g of anhydrous powder was mixed with 1.43 g of deionized water for the cement paste (w/b ratio of 0.4). The pastes were mixed first by hand in an ampoule (external mixing) and then were directly placed in the isothermal calorimeter. Quartz glass was used as the reference material.

### 2.4. X-ray Diffraction and Thermogravimetric Analysis

An organic exchange method was conducted to stop the hydration of all paste specimens. First, the coarse-paste specimens (1.18–2.36 mm) were immersed in isopropanol for 30 min and then in fresh isopropanol for an additional 6 h. The paste specimens after the organic exchange were dried in a vacuum desiccator under ~0.08 MPa for 3 days. The coarse-paste specimens were crushed in an agate mortar, then, the paste powder was passed through a 96-μm sieve. The powders were analyzed by X-ray diffraction (XRD) (D/MAX-2500, Rigaku, Japan) with a Cu-Kα radiation of 1.541 Å, step width of 0.02° and scanning speed of 4°/min at 45 kV and 40 mA. The TGA was conducted by an equipment (DTG-60, Shimadzu, Japan) with a N_2_ purge of 20 mL/min at the heating rate of 10 °C/min from 40 to 1000 °C.

### 2.5. Chloride Binding Capacity

The chloride binding capacity of the paste specimens at 28 days were evaluated using an equilibrium method, using crushed samples trapped between 1.18 and 2.36 mm of a sieve. The equilibrium method was conducted in the NaCl solutions. The crushed samples were immersed in 0.05, 0.1, 0.25, 0.5, 1.0 and 3.0 M solutions of NaCl with a solution-to-solid ratio of 10 (2 g of cement paste and 20 g of chloride solution) at 20 ± 1 °C. Measurements of the NaCl solutions were conducted after 2 months to allow enough time for equilibration in the solutions, as reported in some researches [10,11,28,29]. For potentiometric chloride titration, the equilibrated solutions were pipetted by 0.5 mL for dilution with distilled water. The diluted solutions (~200 mL) were treated before the chloride titration using 1 mL of diluted nitric acid and 1 mL of hydrogen peroxide to reduce the interference with sulfate and hydroxide ions. A potentiometric titrator (Metrohm Titrator 905, Metrohm, Herisau, Switzerland) was used for chloride titration with 0.01 M of AgNO_3_ solutions. Three different samples for each type were measured to check the reproducibility.

The bound chloride content and equilibrated concentration of the chloride solution were calculated according to Equations (1) and (2):

C_b_ = (C_i_ − C_f_) × (1000 × 35.45 × V_L_)/m, (1)

C_f_ = (V_Ag_ × C_Ag_ × M_Cl_)/v. (2)

here, C_b_ is the bound chloride content (mg Cl/g paste), C_i_ is the initial concentration of chloride in the solutions (mol/L), C_f_ is the concentration of the equilibrated chloride solution (mol/L), V_Ag_ is the volume of AgNO_3_ consumed for chloride titration (L), C_Ag_ is the concentration of AgNO_3_ for chloride titration (0.01 mol/L), M_cl_ is the molar mass of chloride (35.45 g/mol), m is the weight of cement paste in the chloride solution (2 g), V_L_ is the initial volume of the chloride solution (20 mL) and v is the volume of the chloride solution extracted from the equilibrated solution (0.5 mL).

The chloride binding capacity was evaluated by the adsorption isotherm (Langmuir, Freundlich and Brunauer-Emmett-Teller (BET) adsorption isotherm). Most research has investigated the chloride binding capacity of cement hydrates using the Langmuir isotherm and Freundlich isotherm [28,29] but the most general form of BET adsorption isotherm has been additionally applied to determine the chloride binding behavior in macroporous materials (1.18–2.36 mm of paste was used in this study). The adsorption isotherm will be discussed in more detail in Section 3.2.

A part of the immersed samples in the NaCl solutions were investigated to determine the factor affecting the chloride binding capacity. The crushed pastes immersed in 0.05, 0.5 and 3 M chloride solutions were analyzed using XRD and TGA, as described in Section 2.4, which can identify the change in the cement hydrate composition. In particular, the approximate quantification of the AFm compounds was implemented by measuring the bound water of the main layers, [Ca_4_Al_2_(OH)_12_]^2+^, comprising hydroxyl groups by TGA analysis [30,31]. As AFm compounds are generally regarded as chemically bound chlorides, the physically bound chlorides could be calculated by subtracting the chemically bound chloride content from the total bound chloride content [32,33]. Thus, the influence of chemical and physical chloride binding on the chloride binding capacity can be approximated by varying the chloride concentration.

### 2.6. Mercury Intrusion Porosimetry

Mercury intrusion porosimetry (MIP) was conducted using porosimetry equipment (AutoPore IV 9500, Micromeritics, USA) to analyze the porosity and pore size distribution of the mortar specimens. Because the MIP method is influenced by the sample preparation process, a sample preparation method was considered with reference to the previous research [34]. After 56 days (water cured for 55 days), the mortar specimens were cut into rectangular prisms (maximum value of the smallest dimension was less than 5 mm) using a micro cutting machine. The prism specimens were first immersed in isopropanol for 7 days at 20 ± 1 °C and then dried at 90 mbar in a vacuum container for 4 h. Approximately 3.5 g of all specimens were used for each measurement (each type of specimen was measured twice). The operating conditions were set as 480 mN/m of mercury surface tension, 130° of mercury-solid contact angle, 10 s of equilibration time and 0.54–33,000 psi of pressure.

### 2.7. Chloride Migration and Natural Diffusion Test

An evaluation of the chloride penetration was conducted by the non-steady state migration (NSSM) test and the natural diffusion test. In this study, all mortar specimens for the chloride migration and natural diffusion tests were cured for 55 days in water after de-molding to reduce the aging effect. The NSSM test was conducted in accordance with the Nordic standard NT Build 492 [35]. The cylindrical mortar specimens (ø100 mm × 50 mm) were obtained by cutting the specimens of ø100 mm × 200 mm. Three specimens in each of PM, AM and CM were used for the NSSM test. For the catholyte solution, 10% of NaCl solutions were used, while 0.3 M of NaOH solutions were used for the anolyte solution. The initial current was identified by applying 30 V in all specimens and was adjusted to the recommended guideline. The currents were recorded for test durations of 24 h for all tests. After the NSSM test was complete, axial splitting was performed for the cylindrical specimens and the 0.1 M AgNO_3_ solution was sprayed onto the rough cross section to determine the chloride penetration depth. The chloride migration coefficient (D_NSSM_) was calculated using the simplified Equation (3) based on the Nernst-Planck equation [35]:

*D_NSSM_* = (0.0239 × (273 + T) × L)/(U − 2) × t × (x_d_ − 0.0238 × √(((273 + T) × L × x_d_)/(U − 2))). (3)

here, *D_NSSM_* is the chloride migration coefficient (×10^−12^ m^2^/s), T is the average temperature between the initial and final temperatures in the anolyte solution (°C), L is the height of the cylindrical specimen (mm), t is the test duration (h) and U is the applied voltage (V).

A natural chloride diffusion test was conducted in accordance with NT Build 443 [36]. The cylindrical mortar specimens of ø100 mm × 70 mm were used for the natural diffusion test, otherwise the NSSM test was conducted. The specimens were coated with epoxy resin, except the one exposed surface and then were immersed in solutions of 165 g of NaCl per dm^3^ for 60 days at 20 ± 1 °C. After immersion, the specimens were kept in a sealed plastic bag for 4 weeks. The author expected that the chloride diffusion would appear from the chloride-limited source and that it would exhibit an influence of the chloride binding behavior on the chloride diffusion more clearly. The specimens were sliced according to the thickness (3, 5, 5, 5, 5 and 5 mm) from the surface and dried at 105 ± 1 °C. The dried mortar samples were grinded by hand in an agate mortar and passed through a 150-μm sieve for acid-soluble and water-soluble chloride determination. Mortar powders of 1 g with 0.1-mg precision were used for the acid-soluble and water-soluble chloride test. The acid-soluble chloride and water-soluble chloride content in a mortar are considered as the total chloride and free chloride, respectively.

For the acid-soluble chloride test, the mortar powder was treated with 10 mL of the diluted nitric acid in a conical tube for 10 min with shaking and 3 mL of hydrogen peroxide (35%) was injected into the solutions to remove the sulfur. The conical tubes were heated at 55 ± 1 °C in a water bath for 5 min after the addition of 30 mL of distilled water. The heated solution was then filtrated using filter paper with a vacuum pump. Then, the filtrated solutions were diluted with distilled water by the solutions of 200 mL.

Nitric acid was used for the first treatment in the acid-soluble chloride test, while distilled water was used for the first treatment in the water-soluble chloride test. The mortar powder was treated with 40 mL of distilled water in a conical tube for 10 min with shaking and was heated at 55 ± 1 °C in a water bath for 5 min. The heated conical tube was kept in the room temperature condition for 1 day to stabilize the solution. The solutions were then filtrated using filter paper with a vacuum pump and diluted with distilled water by the solutions of 200 mL.

All the treated solutions for the acid-soluble and water-soluble chloride tests were treated with an additional 1 mL of the diluted nitric acid and 1 mL of the hydrogen peroxide once more before the potentiometric chloride titration. The measured chloride mass in the solution by the chloride titration was divided by the dried mortar mass (1 g) to calculate the acid-soluble chloride content (wt % of dried mortar) and the water-soluble chloride content (wt % of dried mortar). Notably, the acid-soluble chloride content was regarded as the total chloride content to be used for calculation of the apparent diffusion coefficient.

Fick’s 2nd law is commonly used to describe the natural chloride diffusion in a porous cement composite. The shape of the error function [37] or Gaussian function [38] can be used to calculate the diffusion coefficient, which is dependent on the environment of chloride penetration, such as the relative humidity, saturation degree in a cement composite and the interaction between the chloride ions and cement hydrates. The total chloride content C(x,t) (wt % of dried mortar) in a mortar at depth x (m) and exposure time t (s) can be calculated using the inverse error function erfc [37], given in Equation (4) and the half-Gaussian diffusion model [38], given in Equation (5).

C(x,t) = *C_s_* × erfc(x/(2√(*D_a_* × t))). (4)

here, *D_a_* is the apparent chloride diffusion coefficient (×10^−12^ m^2^/s), *C*_s_ is the total chloride content at the surface. The initial chloride content from the mixtures is ignored because it was very low and not critical for the fitting.

C(x,t) = *M*/(2√(π × *D_i_* × t)) exp(−x^2/(4*D_i_* × t)) = *m*/√(π*D_i_* × t) exp(−x^2/(4*D_i_* × t)). (5)

here, *D_i_* is the chloride diffusion coefficient (×10^−12^ m^2^/s) at the instantaneous limited supply of chloride ions, *M* (wt % of sample) is the total amount of diffused chloride ions in the Gaussian diffusion model (two-way diffusion model), *m* (=*M*/2) (wt % of sample) is the total amount of diffused chloride ions in the half-Gaussian diffusion model (one-way diffusion model). The half-Gaussian diffusion model [38] is used to support erfc and evaluation of the limited chloride diffusion as the specimens were kept in a sealed plastic bag for 4 weeks. It should be noted that all the fittings for chloride diffusion were conducted by applying a relative weighting to the dependent values (weighting by 1/*σ_y_*^2^) because the large values contribute more to the sum of squares in the residuals and thus dominate the curve fittings. In all fittings by the error function and half-Gaussian equation, the measured chloride content in the outermost layer was ignored.

## 3. Results and Discussion

### 3.1. Heat of Hydration

**Figure 1:**
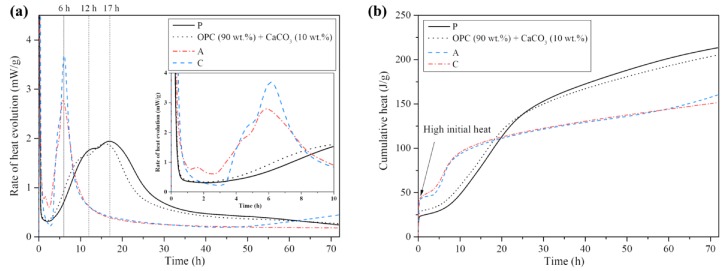
Heat of hydration of P, A and C for 72 h: (**a**) Rate of heat evolution; (**b**) Cumulative heat.

The rate of heat evolution (mW/g) and cumulative heat (J/g) for up to 72 h for the three types of pastes are given in Figure 1. The maximum rate of heat evolution after the initial stage (0–2 h) was observed at approximately 17 h in paste P but the maximum rates of heat evolution in pastes A and C were identified at approximately 6 h, as shown in Figure 1a. The cumulative heat in pastes A and C after 20 h was lower than that in paste P, while pastes A and C showed higher initial heat values within 1 h than paste P, as shown in Figure 1b. The heat evolution of the binder (composed of OPC (90 wt % of binder) and CaCO_3_ (10 wt % of binder) with the same w/b ratio) was measured to determine the influence of calcite on the hydration reaction (dotted line in Figure 1).

The high initial heat within 1 h was mainly due to the ettringite formation from the aluminate reaction with sulfate [39,40]. In the OPC-based binder blended with HAC, C_3_A (3CaO·Al_2_O_3_), CA and CA_2_ could be dissolved as it participates in the initial stage. Nehring et al. [40] reported that the ettringite precipitation appeared very quickly due to the dissolution of CA rather than C_3_A in the OPC paste blended with aluminate cement comprising 40.2 wt % Al_2_O_3_.

After the initial stage, the broad second peak and the third peak caused by calcium silicate hydrate (C-S-H) and Ca(OH)_2_ [39,40] were identified in the acceleration stage of P. The peaks are shown in various types according to the alkali content and mineral composition of OPC [41]. The equivalent alkali content (Na_2_O + 0.658·K_2_O) according to ASTM C 150 [42] was 0.837%, as shown in Table 1. The higher third peak, compared with the second peak, in paste P might be appeared due to the high equivalent alkali content [41,43] and low tricalcium silicate (C_3_S) content [41].

**Figure 2:**
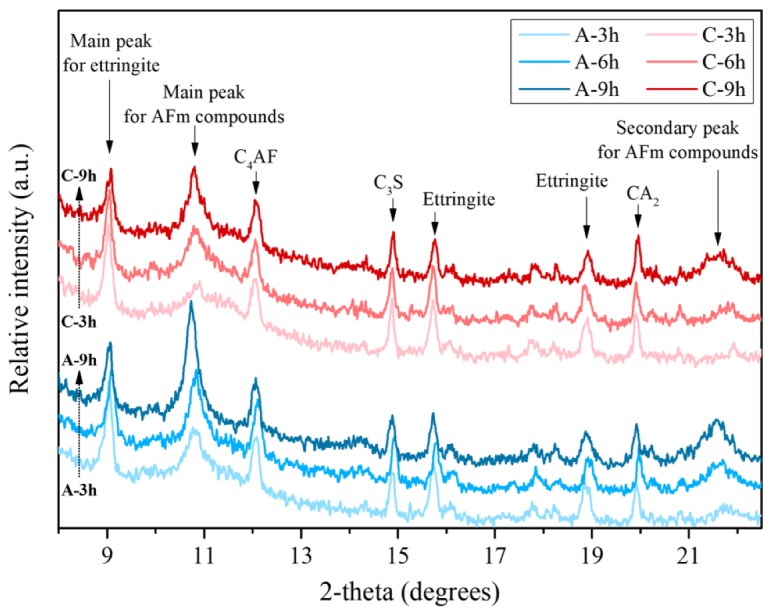
X-ray diffraction (XRD) patterns of A and C at 3 h, 6 h and 9 h after the start of hydration; brownmillerite (tetracalcium aluminoferrite, C_4_AF), Alite (tricalcium silicate, C_3_S).

The sharp peak (acceleration stage) appeared quickly at approximately 6 h in A and C. Furthermore, the XRD analysis was conducted to determine the reason for the quick sharp peak. Figure 2 displays the XRD results of A and C at 3, 6 and 9 h after the hydration reaction. The relative intensity changes in the ettringite and AFm compounds peaks could be identified and are given in Table 3. In addition, we could determine the area of the main peak in each ettringite and AFm compounds. From 3 h to 6 h, the ettringite gradually decreased, while the AFm compounds increased. Paste A showed a higher AFm compound-to-ettringite ratio than paste C. As the OPC (sulfate source) content in A could not be less than that in C, the ettringite content as well as the AFm compounds in A were higher than those in C according to the peak area.

A high alumina source content can lead to CAH_10_ and/or C_2_AH_8_ [44,45]; thus, will cause the rapid consumption of gypsum, anhydrite and basanite with them [45]. This leads to ettringite formation, which converts to AFm compounds after sulfate exhaustion. The sharp hydration peak of C_3_A appeared early due to the lack of sulfate, which was consumed with CAH_10_ and/or C_2_AH_8_ [46]. The high and sharp peak in A, compared with C, between 4 h and 8 h may be caused by OPC hydration and/or the production of more ettringite and AFm compounds in A due to the high sulfate content (theoretically more OPC clinkers and sulfate are present in A than in C). In particular, CaCO_3_ in C appears to act as a nucleation seed [25,26] for growth of hydration products, which can accelerate the hydration process. because the rate of heat evolution between 1 h an 5 h in C is higher than that in A. The OPC substituted with 10% of CaCO_3_, dotted line in Figure 1a, also shows a faster set than of paste P. CaCO_3_ can also increase the stability of ettringite [26], hence, the conversion to AFm compounds can appear less in C than in A.

The post-acceleration stages in A and C appear early, compared with that in P. This stage is related to the further hydration of C_3_S and β-dicalcium silicate (β-C_2_S) [23]. The early post-acceleration stages with low heat rates in A and C can affect the delayed silicate reaction, which might be due to the production of a protective layer on the unhydrated clinker grains [44]. Therefore, a difference in the average compressive strength (see Section 2.2) among the mortars (PM, AM and CM) appeared after 56 days due to less silicate reaction.

### 3.2. Chloride Binding Capacity: Chloride Binding Isotherm

**Figure 3:**
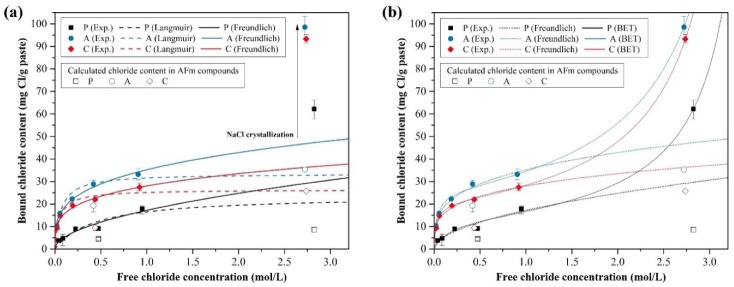
Chloride binding isotherms of P, A and C: (**a**) Langmuir and Freundlich isotherms; (**b**) Freundlich and Brunauer-Emmett-Teller (BET) isotherms.

Figure 3 displays the bound chloride content (C_b_, mg Cl/g paste) of pastes P, A and C against the equilibrated chloride concentration (C_f_, mg Cl/L). The experimental data in Figure 3a is fitted by the Langmuir and Freundlich isotherms [47], Equations (6) and (7), respectively, as follows:

C_b_ = (*C_max_* × *K_L_* × C_f_)/(1 + *K_L_* × C_f_). (6)

here, *C_max_* is the maximum amount of adsorbed chloride on the adsorbent (mg Cl/g paste) and *K_L_* is the Langmuir isotherm constant (L/mg Cl);

C_b_ = *K_F_* × (C_f_)^(1/*n*). (7)

here, *n* is an experimental constant representing the adsorption intensity and *K_F_* is the Freundlich isotherm constant ((mg Cl/g paste) (mg Cl/L)^n^).

Langmuir adsorption assumes that the adsorbates are adsorbed on the homogeneous surface with no interaction between the adsorbates whose distinctive property reveals a horizontal plateau as the adsorbate content increases continuously. However, Freundlich adsorption assumes adsorption on a heterogeneous surface and multilayer adsorption, which can reveal the gradual accumulation of adsorbates on the surface from the competitive adsorption as the adsorbate content increases continuously [47]. Therefore, it is suggested that the chloride adsorption on cementitious materials obeys Langmuir adsorption at low concentrations of less than 0.05 M of chloride and Freundlich adsorption at high concentrations, in the report by Tang and Nilsson [28]. At lower adsorbate concentrations, monolayer adsorption begins first and multilayer adsorption occurs as the concentration increases. Most research has shown that the Freundlich form was a better fit for determining the chloride binding isotherm within 1–2 M chloride concentrations [9,17,18,28,48]. As the majority of research considered the chloride concentration range of 1–2 M NaCl solutions, which covers the chloride concentration of seawater (~0.5 M), it can be reasonable in a general marine environment, except a special environment affected by a de-icing salt. Thus, the chloride binding behavior of samples P, A and C can be estimated using the Freundlich form.

The experimental data for 3 M of chloride solution was excluded for fitting by the Langmuir and Freundlich isotherms. As the bound chloride steeply increased for chloride concentrations higher than 1 M, the adsorption isotherm would overestimate the bound chloride content in the chloride concentration range of 1–3 M if the data of 3 M of chloride solution is included in the fitting process. This is the reason that we introduced the BET adsorption form to describe the chloride binding behavior in this study. Furthermore, this should be investigated because the NaCl solution of 165 g/L (~2.8 M) was used in the natural chloride diffusion test. The previous research [49] provides a comparison between Freundlich’s equation and the modified BET equation, suggesting that the chloride binding behavior can differ above 1 M in a NaCl solution. Several solids, such as clay, pigments and cement, can lead to Type II adsorption according to the International Union of Pure and Applied Chemistry (IUPAC) classification of isotherms, for unrestricted monolayer-multilayer adsorption [47]. As ion clusters are observed between 1 M and 5 M in NaCl solutions [50], we can suppose that the NaCl interaction should not be ignored at higher chloride concentrations. Although the adsorbate interaction is not sufficiently described [47], the BET form can explain the unrestricted monolayer-multilayer adsorption [47]. The experimental data in Figure 3b was fitted by the BET isotherm as follows:

C_b_ = (*C_m_* × *B* × (C_f_/*C_s_*))/[1 − (C_f_/*C_s_*)][1 + (*B* − 1) × (C_f_/*C_s_*)]. (8)

here, *C_m_* is the monolayer adsorption capacity of chloride (mg Cl/g paste), *C_s_* is the saturation chloride content (mol/L) and *B* is the BET isotherm constant related to the enthalpies of adsorption between the first and higher layers. The most general form of the BET equation uses the relative pressure and the adsorption occurs according to the pressure gradient. Herein, however, the relative concentration (C_f_/C_s_), as the concentration gradient, was used as a driving force, which corresponds to the pressure gradient.

Table 4 shows the experimental parameters of the adsorption isotherm equations for all samples computed by the nonlinear fitting. The Freundlich form provided a better fit than the Langmuir form in the range of 0.0–1.0 M of NaCl solutions, considering the adjusted determination of the coefficient (adjusted R-squared). The BET form displayed a better fit for all ranges of NaCl solutions compared with the Langmuir and Freundlich forms.

In the experimental parameters of the Freundlich form, the parameter n represents the binding intensity, which affects the shape of the fitted curve in which the bound chloride content increases steeply at lower chloride concentrations. As the parameter n in P is much lower than those in A and C, the bound chloride in P increased gradually below the chloride concentration of 0.5 M. The parameter *K_F_* relates to the binding capacity; here, *K_F_* in P was also much lower than those in A and C, indicating that the chloride binding capacity was much lower than those in A and C. However, care should be taken when interpreting the parameters because n also affects the bound chloride content, as shown in Equation (7); thus, the more that n decreases, the more the binding capacity increases. This explains why *K_F_* in A is smaller than that in C, unlike that shown in Figure 3a.

Although the Freundlich form can explain well for chloride concentrations below 1 M, we investigated the adsorption isotherm for describing the binding behavior for chloride concentrations up to 3 M. The bound chloride content that increased steeply for chloride concentrations above 1 M can be fitted well with the BET form for the adsorption isotherm, as presented in Figure 3b. Furthermore, the BET form provides a better fit for the wide range of chloride concentrations in this study, though the chloride binding behavior has not been clearly described at high NaCl concentrations above 2 M in previous research. This might be because unrestricted monolayer-multilayer adsorption is dependent on a macropore at high concentrations of NaCl solutions. The estimated monolayer adsorption capacities by the BET form are 13.52, 25.68 and 20.72 mg Cl/g paste in P, A and C, respectively. After the monolayer adsorption occurs, the adsorbed chloride exponentially increases in the BET form. However, only we can describe the chloride binding behavior within the saturation chloride content because we defined the maximum chloride concentration to replace the relative pressure in Equation (8).

In summary, the chloride binding capacities of P, A and C were compared using the Langmuir, Freundlich and BET adsorption isotherm forms. The chloride binding capacity followed the order A > C > P for all ranges of chloride concentration, except below approximately 0.01 M. Although it was difficult to describe the chloride binding behavior between the chloride concentrations of 1 and 3 M, we found that the chloride binding capacity followed the order A > C > P until the chloride concentration reached 3 M. Recent research [51] has shown using X-ray fluorescence that the bound chloride content steeply increased between the NaCl concentrations of 1 and 3 M, which seemed to be more discussed. The Freundlich and BET forms could be used to describe the chloride binding behavior below the chloride concentration of 1 M because they were similar, as shown in Figure 3b. The BET form showed similar binding behavior as that in the Freundlich form until the chloride concentration reached 1 M in A and C and 1.5 M in P. Thus, we could conclude that the monolayer adsorption capacity from the BET form was more reasonable than that from the Langmuir form.

### 3.3. Chloride Binding Capacity: X-Ray Diffraction and Thermogravimetry

**Figure 4:**
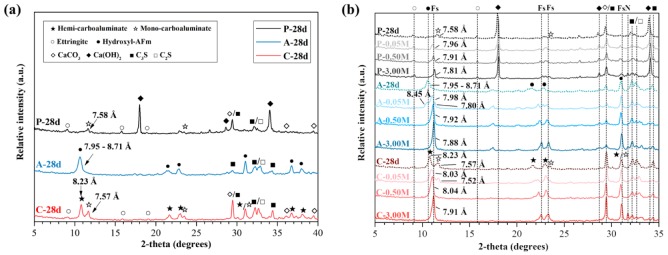
XRD patterns of P, A and C (**a**) at 28 days; (**b**) chloride contaminated samples with 0.05, 0.50 and 3.00 M chloride concentrations in which ★: hemi-carboaluminate, ☆: mono-carboaluminate, ○: Ettringite, ●: hydroxyl-AFm, ■: tricalcium silicate (C_3_S), □: β-dicalcium silicate (β-C_2_S), ◇: CaCO_3_, ◆: Ca(OH)_2_, Fs: Friedel’s salt and N: NaCl.

**Figure 5:**
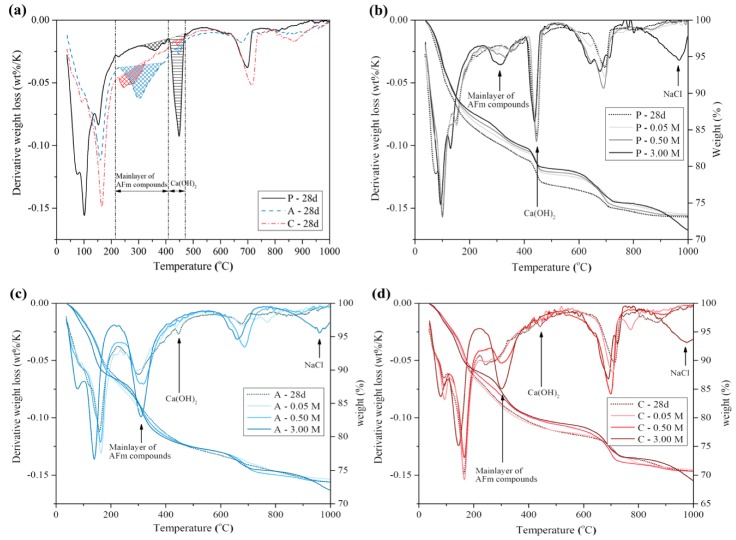
Thermogravimetric analysis (TGA) results of P, A and C (**a**) at 28 days; chloride contaminated samples (**b**) P, (**c**) A and (**d**) C at 0.05, 0.50 and 3.00 M chloride concentrations.

Figure 4 displays the XRD patterns of the paste samples at 28 days and the chloride contaminated samples used in the measurement of the chloride binding capacity. Figure 5 displays the TGA results of the samples as the same as those used for the above XRD measurement. Large differences of the samples at 28 days include the relative intensity of AFm compounds and Ca(OH)_2_, as shown in Figure 4a. The XRD patterns of the AFm compounds in A and C were clearly identified between the 2θ values of 10° and 12° and the main peak of Ca(OH)_2_ was found at 2θ of approximately 18°. The main peaks of C_3_S, β-C_2_S and CaCO_3_ were also identified between 2θ of 29° and 35°. In the Figure 4b, the change in the basal peaks of AFm compounds after the chloride contamination with the different chloride concentrations of 0.05, 0.5 and 3.0 M could be found. Additionally, the peaks of unhydrated C_3_S and β-C_2_S in A and C were more noticeable than the that in P. It can be caused due to the delayed silicate reaction [44] as shown in Figure 1.

As the calcite and gypsum in Portland cement can react in the hydration process, the anions (OH^−^, SO_4_^2−^ and CO_3_^2−^) and water molecules are expected to combine in the interlayer of the AFm compounds. In particular, the interplanar spacing of the basal peaks in the AFm compounds are different according to the interlayer anion composition [52,53,54]. Thus, we can determine the type of AFm compounds based on the basal peak position and the interplanar spacing. Figure 4a displays the basal peak in the AFm compounds of the samples. The computed interplanar spacing was compared with the previous research of Matschei et al. [52]. The XRD pattern of P at 28 days shows the basal peak of mono-carboaluminate, which reveals an interplanar spacing of 7.6 Å. The XRD pattern of A at 28 days shows the basal peak of hydroxyl-AFm type, revealing an interplanar spacing of 7.95–8.71 Å. The XRD pattern of C at 28 days shows the basal peak of hemi-carboaluminate and mono-carboaluminate, which reveals interplanar spacings of 8.2 Å and 7.6 Å, respectively. The effect of the amount and the type of AFm compound on the chloride adsorption are addressed in the following XRD and TGA results.

The change in the basal peaks was determined based on the change in the interplanar spacing. According to the research of Balonis et al. [53], the interplanar spacing of the basal peak in a chloride-bearing AFm compound is revealed to range from 7.8 Å to 7.9 Å.

All the samples at 28 days were gradually transformed to chloride-bearing AFm compounds as the chloride concentration increased to 3.0 M. As well as the transformation, an increase in the relative intensity of chloride-bearing AFm compounds was identified, particularly in A and C. The AFm compounds in P and A at 28 days were almost transformed to chloride-bearing AFm compounds at approximately 0.5 M chloride solution, however, the AFm compounds in C at 28 days were almost transformed above 0.5 M chloride solution according to the interplanar spacing of the basal peak in the chloride-bearing AFm compounds. This appears to be influenced by the type of AFm compound. The carbonates are efficiently packed in mono-carboaluminate, compared with those in hemi-carboaluminate, while the interplanar spacing of the basal peak in mono-carboaluminate is smaller than that in hemi-carboaluminate [53]. The densely packed structure might have a higher stability than that less densely packed. Although the structural property is important, the chloride-to-solid ratio is also important because the relative amount of NaCl solution can affect the stability of the AFm compounds [20]. Thus, the low amount of mono-carboaluminate in P might be easily transformed to chloride-bearing AFm compounds.

The amount of AFm compound was calculated using the TGA results. Figure 5a shows the TGA results of the samples at 28 days. The hydrate content at the specified range in which H_2_O or CO_2_ was released were also calculated; those of the paste samples are shown in Table 5. We will discuss further the amount of AFm compound and bound chloride content in the AFm compounds, which is generally regarded as chemically bound chloride [9]. Determining the amount of AFm compounds is quite complicated because the weight loss region of the AFm compounds is different depending on the type of AFm compound [31]. Herein, the AFm compounds content at 28 days and 0.5 M chloride solution is approximated, however, the AFm compounds content at 3 M chloride solution was calculated by assuming that all AFm compounds are Friedel’s salt. To calculate the Friedel’s salt content according to Equation (9), the derivative weight loss in the range of 230–410 °C was used for the loss of the main-layer water (6·H_2_O) [20,31]. Tangential integration was used with a straight baseline for the loss of the main-layer water.

W_Fs_ = (M_Fs_/6M_w_) × A_w_. (9)

here, *W_Fs_* is the Friedel’s salt content in the sample (wt % of sample), *M_Fs_* is the molar mass of Friedel’s salt (561.34 g/mol), *M_w_* is the molar mass of a water molecule (18.02 g/mol) and *A_w_* is the integrated area in the range of 230–410 °C [30,31]. The chloride content in Friedel’s salt was calculated by multiplying the mass fraction of 2 mol chloride to 1 mol Friedel’s salt.

Similar to the Friedel’s salt calculation, it was assumed that the six main-layer water in mono-carboaluminate (568.29 g/mol), hemi-carboaluminate (564.45 g/mol) and hydroxyl-AFm (560.47 g/mol) were lost for 230–410 °C [31]. However, the straight baseline for the integration area was considered flexibly in the tangential integration. The AFm content of the chloride contaminated samples in 0.05 M chloride solutions is not considered because the interlayer anions of the AFm compounds are quite complicated, compared with the AFm compounds in high concentrations of chloride, in which the AFm compound is assumed as a Friedel’s salt. Although the interlayer anions cannot fully comprise chloride anions below 3 M chloride solutions [55], the transformation into Friedel’s salt is also dependent on the molar ratio of chloride-to-AFm compounds. Mesbah et al. [54] reported that Friedel’s salt, rather than Kuzel’s salt, was controlled above the chloride-to-monosulfoaluminate molar ratio of 3. Herein, the chloride-to-AFm compounds molar ratio at 0.05 M is approximately less than 3 based on the TGA results and the experimental conditions (water-to-solid ratio of 10), however, it is much higher than 3 for the chloride solutions of >0.5 M. Therefore, we can assume that the chloride amount in the interlayer space, [xCl^−^·((2 − x)/n)X^n−^] (with X^n−^ = SO_4_^2−^, CO_3_^2−^ and OH^−^), is between 1.5 < x < 2.0 for the 0.5 M chloride solutions. The molar mass of the AFm compounds (chloride amount of 1.5 < x < 2.0 in the interlayer) was calculated by addition or subtraction of the anion molar mass from the molar mass of Friedel’s salt for calculation of the bound chloride in the AFm compounds, as shown in Table 5. The interplanar spacing results, shown in Figure 4b, are also considered to support the above assumption, as well as the previous research [53]. Thus, for the calculated chloride content in the AFm compounds at 0.5 M chloride solutions, the chloride content range in the AFm compounds is regarded as the chloride content between the interlayer chloride amount of 1.5 < *x* < 2.0. Additionally, the Ca(OH)_2_ content and bound water content were calculated to check the hydration change. The Ca(OH)_2_ content was calculated using a tangential method based on a straight line [31]. Meanwhile, the bound water content was calculated by dividing the weight loss of samples from 50 to 550 °C by the sample weight at 550 °C [56].

Table 5 shows the TGA results of the paste samples at 28 days and the chloride contaminated samples in the 0.05, 0.5 and 3 M chloride solutions using the TGA data are shown in Figure 5b–d. The Ca(OH)_2_ and bound water content in all samples decreased slightly as the chloride concentration increased. The estimated AFm content also increased compared with the initial AFm content at 28 days as the chloride concentration increased. The average calculated chloride content in the AFm compounds in the 0.5 M chloride solutions were 4.5, 19.3 and 9.4 mg Cl/g sample for samples P, A and C, respectively and the calculated chloride content in the 3 M chloride solutions were 8.6, 35.3 and 25.8 mg Cl/g sample, respectively.

**Figure 6:**
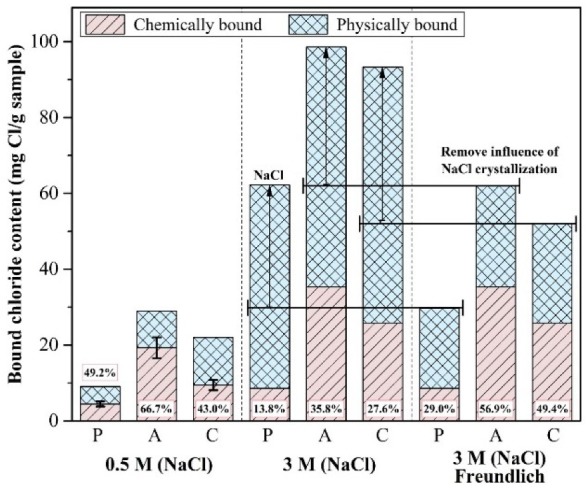
Calculated chemically bound chloride content and its composition regarding the total bound chloride content.

The contribution of these bound chlorides is shown in Figure 6. In addition, the effect of the AFm compounds on the chloride binding capacity can be calculated by the weight percent of the total bound chloride. The bound chloride, including an amount of crystallized NaCl, is regarded as the total bound chloride on the left side, whereas, when calculated by the fitted Freundlich equation, it is regarded as the total bound chloride on the right side. The amounts of 49.2 ± 7.0%, 66.7 ± 9.5% and 43.0 ± 6.1% of the total bound chloride in the 0.5 M chloride solution are chemically bound chloride, when considering the maximum and minimum value. Although the crystallization of NaCl was measured as a bound chloride (left side) in the 3 M chloride solution, it was not a strictly physically bound chloride of the cement hydrates. Thus, the total bound chloride in the 3 M chloride solutions was corrected (right side) using the fitted Freundlich equation, given in Table 4.

The contribution of chemically bound chloride for the chloride adsorption in P decreased significantly in the 3 M than 0.5 M chloride solution, when it compared with the contribution of physically bound chloride. However, the contribution of chemically bound chloride in A and C slightly decreased or was similar, when it compared with results of 0.5 M chloride solution. The chemically bound chloride and physically bound chloride can be influenced by the AFm compounds content and the specific surface area respectively [32]. The influence of the physical chloride adsorption can increase as the chloride concentration increases according to the results of previous research [32,33]. If the chemical source (e.g., CaO, Al_2_O_3_) for the layered double hydroxide generation is low, the generation of AFm compounds is limited. As the layered double hydroxides theoretically have a limited chloride binding capacity [57], the effect of chemically bound chloride can be reduced for high chloride concentrations, whereas, the physically bound chloride can affect the chloride binding capacity more. However, if the AFm compounds content is relatively high it can affect the chloride adsorption more in the high chloride concentration. Thus, it might be higher contribution of chemically bound chloride in A and C until the 3 M chloride solution because we cannot ignore the AFm compounds content in A and C.

### 3.4. Mercury Intrusion Porosimetry

**Figure 7:**
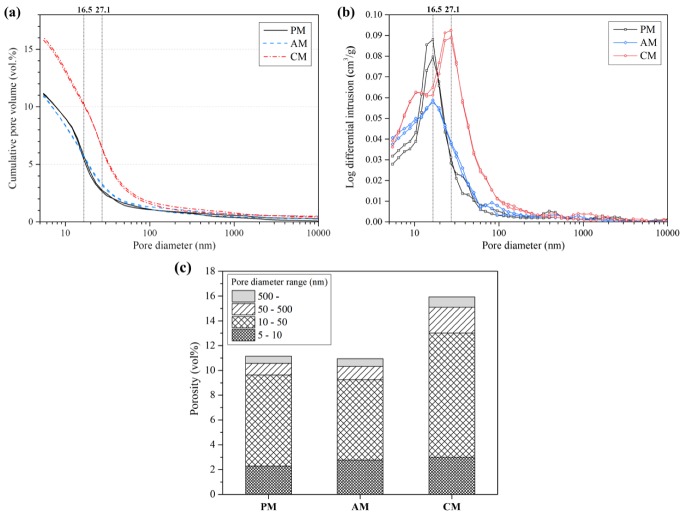
Mercury intrusion porosimetry (MIP) results of the mortar PM, AM and CM: (**a**) Cumulative pore volume; (**b**) Log differential pore volume; (**c**) Porosity according to the pore diameter range.

Figure 7 shows the total pore volume and the pore size distribution of the samples. The total volumes of the PM and the AM were almost similar (~11 vol %) but the CM showed the higher total volume (~16 vol %), as shown in Figure 7a. This difference in pore size distribution is displayed in Figure 7b. The critical pore size was ~16.5 nm in both the PM and the AM but 27.1 nm in the CM. Meanwhile, Figure 7c shows the pore fraction of the specimens. The pore fraction is based on the capillary pore classification according to S. Mindess et al. [58]. The total porosity was 11.1%, 11.0% and 15.9% and the capillary pore volume (above 10 nm) was 8.9%, 8.2% and 12.9% of the PM, AM and CM, respectively. The total porosity of the PM was similar to that of the AM but the capillary pore volume of PM was slightly higher than that of AM. Although the pore size of the AM in 20–50 nm was slightly higher, the pore size volume of the AM in 10–50 nm was less than that of the PM. This shows that the pore size volume was more concentrated in 10–20 nm in the AM than in the PM, as shown in Figure 7b. There was not a significantly negative effect on the pore structure of the AM due to the fast setting, compared with the pore structure of the PM (see Section 3.1).

As the critical pore diameter and capillary pore volume can affect the chloride diffusivity [59], the pore characteristics can have a critical effect on the chloride diffusion. In particular, the OPC substitution with CaCO_3_ can accelerate the hydration (see Section 3.1) and increase the porosity, though the porosity change caused by the partial substitution remains debatable [60]. In particular, the transformation from hemi-carboaluminate to mono-carboaluminate at a later age [24,61] can also induce the increase in the pore volume of the CM because the mono-carboaluminate density is higher than that of hemi-carboaluminate [62]. As the total porosity and capillary pore volume are almost similar in the PM and AM, the chloride binding behavior can be a dominant factor in the chloride diffusion behavior. The influence of the capillary pore volume on the chloride diffusion will be addressed together with the chloride binding capacity in the following section.

### 3.5. Chloride Migration and Natural Diffusion

**Figure 8:**
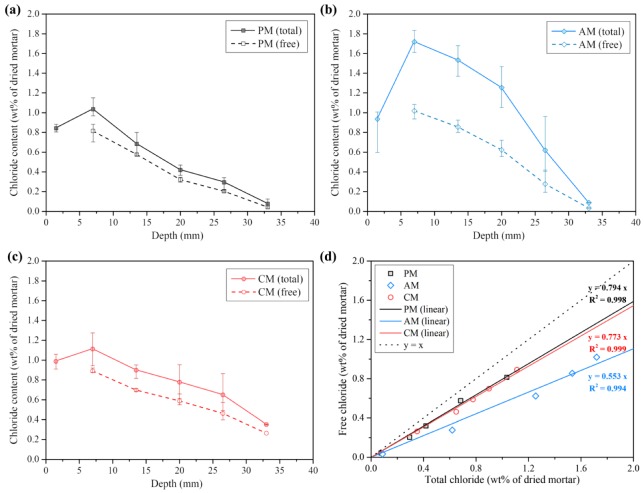
Results of natural chloride diffusion test: total and free chloride content of (**a**) PM, (**b**) AM and (**c**) CM; (**d**) Relationship between the total and free chloride content of the samples.

Figure 8a–c display the results of the natural chloride diffusion test, showing the total chloride content (filled marker symbols) and the free chloride content (open marker symbols). The gap between the total chloride content and the free chloride content represents the bound chloride content at the depth. Figure 8d shows the relationship between the total chloride content and the free chloride content. The free chloride content was followed the order PM ≈ CM > AM according to the total chloride content, which corresponded to the linearity well. Notably, the bound chloride content at the certain free chloride content, unlike the paste samples, was almost similar in the CM and PM. In particular, the AM shows a much higher total chloride content at low depths from the surface compared with the PM and CM. As the high chloride binding capacity can result in the total chloride near the surface due to the driving force in a concentration gradient [63,64], the total chloride content near the surface could be much higher in the AM.

Table 6 shows the experimental results of the chloride migration and natural diffusion tests. The average chloride diffusion coefficients (migration test) of the PM, AM and CM were 19.10, 14.41 and 28.33, respectively. As the migration test is highly related to the pore structures [65,66], the capillary pore volume (see Section 3.4) correlates with the migration test results.

**Figure 9:**
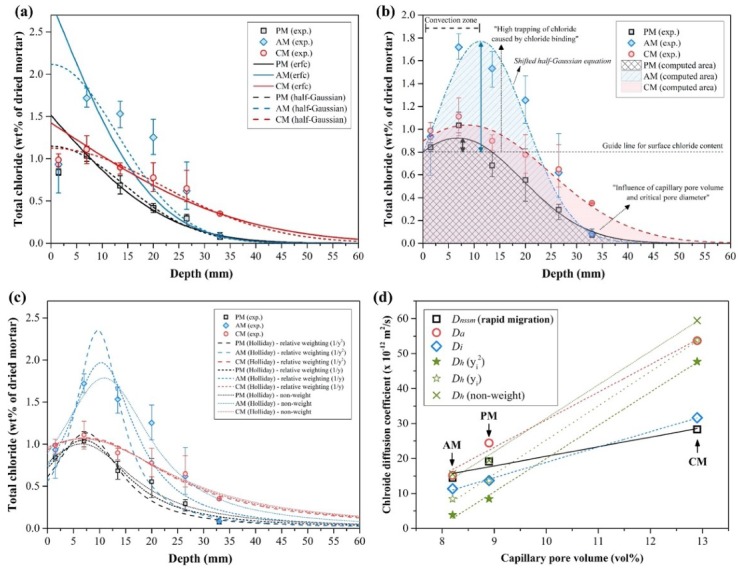
Results of the natural chloride diffusion test and the curve fitting by (**a**) inverse error function and half-Gaussian equation, (**b**) shifted half-Gaussian equation and (**c**) Holliday equation. (**d**) Comparison of the calculated chloride diffusion coefficients between the rapid migration test and the natural diffusion test.

Figure 9a shows the fitted curve by the inverse error function and half-Gaussian equation. All the computed parameters are shown in Table 7. The diffusion coefficients computed by the error function were 24.41, 15.14 and 53.68 for the PM, AM and CM, respectively. Meanwhile, those computed by the half-Gaussian equation were 13.69, 11.35 and 31.66 for the PM, AM and CM, respectively. In all the chloride migration and the natural diffusion results, the chloride diffusion coefficients followed the order CM > PM > AM.

We could clearly identify the convection zone (skin effect) in all samples, particularly in the AM as shown in Figure 9b. In fact, convection and limited chloride diffusion are inevitable in the specimens. The maximum peak of the chloride content can easily appear in the equilibrated specimens at high relative humidity [38]. The fitting by the error function can induce the increase in the computed surface chloride content, which can result in miscalculation of the chloride diffusion coefficient. Therefore, the diffusion coefficients were also estimated using the half-Gaussian equation to consider the limited chloride diffusion. In all specimens, the half-Gaussian equation led to a better agreement with the fitting than the error function. However, the fitting was visibly not good because the maximum peak of the AM in the convection zone was too high. Both fittings could not explain this experimental result in the AM. The shape of the fitting results of the AM appeared similar to that reported in the previous findings. M. A. Climent et al. [38] reported that the half-Gaussian equation could overestimate the chloride content at low depths from the surface but underestimate the chloride content at high depths (above 95% RH condition). Although this seemed incorrect, they compared the diffused chloride amount computed by the half-Gaussian equation with the total experimental amount of chloride content. Accordingly, the diffusion coefficient could be adopted when the diffused chloride amount computed the by half-Gaussian equation and the experimental data (apparent diffused chloride amount) were similar. In this study, the apparent diffused chloride amount was estimated by integrating the shifted half-Gaussian curve, as shown in Figure 9b. The fitted curve is an estimate considering the convection zone based on the experimental data. The actual diffused chloride amount (m_a_) given in Table 8 was computed by integrating the curve fitted by Equation (10) the shifted half-Gaussian equation.

C(x,t) = *m_s_*/√(π*D_i__s_* × t) exp(−*a*(x − *b*)^−2^/(4*D_i__s_* × t)). (10)

In Equation (10), x-squared terms of Equation (5) is shifted. Then, they were compared with the diffused chloride amount (m), a parameter of the half-Gaussian equation. The m/m_a_ ratio of all specimens was almost 1, which could result in a more reasonable diffusion coefficient than that of the error function. We also determined that the higher the chloride binding capacity (see Section 3.3), the higher the amount of diffused chloride in the mortar. This can be due to the driving force in a concentration gradient [63,64].

Additionally, the diffusion coefficient using the Holliday equation (*D_h_*) (Equation (11)) was investigated [67] to support the previous interpretation regarding D_a_ and D_i_. It was proposed to consider the convection zone.

C(x,t) = 1/(*R*_1_ + *R*_2_ × (x − *R*_3_)^−2^) = 1/(*R*_1_ × (1 + (x − *R*_3_)^−2^/(*D_h_* × t))). (11)

here, D_h_ is the chloride diffusion coefficient (×10^−12^ m^2^/s) calculated by the Holliday equation, *R*_1_ (wt % of dried mortar) is the maximum chloride content in the convection zone, *R*_2_ is related to the chloride diffusion coefficient, which corresponds to *R*_1_/*D_h_*·t and *R*_3_ is the distance of the maximum peak from the mortar surface (mm).

The computed parameters and estimated diffusion coefficient using the Holliday equation are given in Table 9. Figure 9c shows the fitted curve using the Holliday equation. Weighted regression was conducted to compensate the extreme value (the lowest value) at the high depth from the surface. If there is no weight, the fitted curve cannot consider the extreme value well. Thus, it was reasonable to use the weighted method (1/*y_i_*^2^ and 1/*y_i_*) for the chloride content (y_i_). Regarding the comparison above of the diffused chloride amount, the fitted curve using the Holliday equation was also integrated to calculate the diffused chloride amount and was compared with m_a_ in Table 8. Although *m_h_*/*m_a_* ranging from 0.90 to 1.10 might be reasonable, it is hard to accept the diffusion coefficient according to this ratio because the extreme value in the non-weight method is not considered. We suggest using the average diffusion coefficient (with/without the weighted regression) in Table 9.

The average diffusion coefficients (Holliday) were lower in the PM and AM but higher in the CM than those calculated from the half-Gaussian. In particular, the average diffusion coefficient (Holliday) was more reduced in the AM than in the PM. However, the average diffusion coefficient (Holliday) of the CM, which was similar to the diffusion coefficient (erfc), increased more than the diffusion coefficient (half-Gaussian). The fitting result by the Holliday equation was similar to the diffusion coefficient (half-Gaussian) in the PM and AM but different to the diffusion coefficient (erfc) in the CM. Although this result requires further investigation, it seems to result from the chloride adsorption and pore characteristics, which might affect the shape of the profile, as shown in Figure 9b. As the chloride binding capacity can affect the chloride diffusion [6,7], increased trapping of chloride ions will appear following the convection zone. The critical pore diameter and the total porosity can also affect the chloride diffusion [68,69]. The pore characteristics might be dependent on the chloride diffusion in the CM, when it is considered that the CM has a higher chloride binding capacity than the PM. The deeper chloride penetration in the CM compared with the PM and AM seems due to the pore characteristics (especially capillary pore volume, see Section 3.4). As shown in Figure 9d, the slope of the linear fit in the natural diffusion test increases compared with that in the rapid migration test (the diffusion coefficient, which is reflected by the interaction between the chloride ions and the cement matrix, has a more positive effect in the order of AM > PM > CM). It seems that the chloride binding behavior in the diffusion process is affected by the capillary pore volume and the capillary pore sizes when the rapid migration test is assumed as almost dependent on the capillary pore volume or pore sizes due to the short duration. We cannot ignore the chloride binding effect for a given pore size distribution, which can induce the trapping of diffused chloride ions (comparison between PM and AM). As the chloride binding is dependent on the electrical double layer on the surface of cement hydrates, the smaller capillary pore size can result in an increase in the chloride diffusion into the surface [70]. This result supports the report of Loser et al. regarding the importance of the pore characteristics rather than the chloride binding capacity for chloride resistance [66].

To summarize, here, we report the chloride resistances of the PM, AM and CM considering the chloride binding capacity of the matrix and the chloride diffusion coefficient. The total and capillary pore volume, critical pore size and chloride binding capacity should be considered for the chloride resistance, as shown in Figure 9b. The chloride diffusion coefficient of the CM where the total and capillary pore volume and the critical pore size are higher than those of the PM is higher than that of PM although the chloride binding capacity of the matrix in CM is relatively high. Therefore, the chloride resistance seems to be mainly influenced by the pore characteristics. Although the physical and chemical chloride binding play an important role in the chloride diffusion, the chloride binding effect can be reduced for a given larger pore size (see Figure 8d). Even for a high chloride binding capacity, it might not significantly contribute to the chloride resistance of cement composites, which are not free from cracking, with large capillary pores or cracks.

## 4. Conclusions

This study aims to determine the chloride resistance of OPC-based mortar blended with HAC and CaCO_3_. The chloride resistance of the PM, AM and CM samples is investigated considering the chloride binding capacity, pore characteristics and the calculated chloride diffusion coefficient. The main results of this study are as follows:

1. The available alumina, which is from the highly reactive calcium aluminate of HAC (15 wt % replacement), can stimulate AFm compounds quickly in the OPC-based system. It is found that the hydration of OPC can be delayed in existence of the calcium aluminate.
2. The chloride binding capacity was higher in order A > C > P. This is mainly due to the proportions of AFm compounds content. The estimated AFm compounds content at 28 days in A and C are 4–5 times more than that in P. Unlike chemical chloride adsorption in P, there is no significant decrease in contribution of chemical chloride adsorption (chloride adsorption by anion exchange in AFm compounds) in A and C as the chloride concentration increases.
3. The capillary pore volume (above 10 nm) are much higher in the CM (12.9 vol %) than in the PM (8.9 vol %) and AM (8.2 vol %). All the estimated chloride diffusion coefficients for the rapid migration and natural diffusion tests are higher in order CM > PM > AM, when they are compared for each fitting method. Although the cement matrix of CM shows a higher chloride binding capacity than that of the PM, the chloride penetration is more active in the CM compared with the PM. Because the capillary pore volume is much correlated to the estimated chloride diffusion coefficients, the effect of chloride binding capacity is limited to reduce the chloride diffusion coefficient. The chloride binding capacity has a positive effect on the estimated diffusion coefficient at small capillary pore volumes. There can be a higher chloride adsorption for a given small capillary pore volume or a critical pore diameter.

Although there have been many studies on the chloride binding capacity, few studies have investigated the chloride binding capacity considering the diffusion behavior. Herein, we determine the most important property affecting the chloride resistance. An increase in the AFm compounds content shows a high chloride binding capacity, as reported in many studies. However, the high chloride binding property cannot only trap the chloride ions near the surface but facilitate the chloride from the outside due to the driving force. From the current study, the chloride binding behavior is expected to be affected by the capillary pore diameter. The critical point might be appeared between the influx of chloride ion and the chloride diffusion from the outside to the inside. Thus, the further investigation of materials with high chloride binding capacity are required varying the different capillary pore volume. We can discuss about the effectiveness of chloride binding capacity compared with the pore characteristics, because the pore characteristics are found to play a highly important role for chloride diffusion.

## Figures and Tables

**Table 1:** Oxide composition of materials (wt % normalized to ~100%).

Chemical Composition [wt %]	Materials		
	OPC	HAC	CaCO_3_
CaO	63.56	29.63	99.75
Al_2_O_3_	5.04	69.42	-
SiO_2_	19.49	0.11	-
Fe_2_O_3_	3.61	0.08	-
MgO	3.28	0.42	-
TiO_2_	0.31	0.02	-
Na_2_O	0.14	0.27	-
K_2_O	1.06	0.01	-
SO_3_	3.38	0.02	-
P_2_O_5_	0.12	0.02	-
Loss on ignition (wt.%)	3.15	0.74	43.85 (CO_2_)

**Table 2:** Mix proportions of specimens.

Specimen Type	Specimen Name	w/b Ratio	Binder Composition [wt %]			Measurement Plan
			OPC	HAC	CaCO_3_	
paste	P	0.4	100	0	0	heat of hydration, chloride binding capacity (chloride potentiometric titration, XRD, TGA)
	A		85	15	0	
	C		75	15	10	
mortar ^1^	PM	0.5	100	0	0	mercury intrusion porosimetry, chloride migration and natural diffusion test
	AM		85	15	0	
	CM		75	15	10	

^1^ Sand-to-binder ratio of mortar specimens is 3. The standard sand was used in accordance with KS L ISO 679 [27].

**Table 3:** XRD patterns of paste A and C at 3, 6 and 9 h after the start of hydration.

Sample		Ettringite (Main Peak)		AFm Compounds (Main Peak)			Peak Area Ratio (AFm/Ett)
		2θ [°]	Relative Area (vs. 3 h)	2θ [°]	d-Spacing [Å]	Relative Area (vs. 3 h)	
A	3 h	9.0	1.00	10.8	8.21	1.00	1.27
	6 h	9.1	0.84	10.8	8.17	1.49	2.24
	9 h	9.0	0.72	10.8	8.23	1.77	3.11
C	3 h	9.0	1.00	10.9	8.15	1.00	0.60
	6 h	9.0	0.95	10.8	8.17	1.65	1.04
	9 h	9.1	0.62	10.8	8.19	2.19	2.12

**Table 4:** Experimental parameters of the adsorption isotherm equations.

Adsorption Isotherm Parameters		Sample		
		P	A	C
Langmuir isotherm	*C_L_* (mg Cl/g paste)	23.71	33.53	26.37
	*K_L_* (L/mg Cl)	2.29	15.91	19.83
	Adjusted R-squared	0.853	0.954	0.935
Freundlich isotherm	*n*	1.903	3.530	3.892
	*K_F_* (mg Cl/g paste)/(mol/L)*^n^*	17.20	35.21	28.02
	Adjusted R-squared	0.900	0.980	0.975
BET isotherm	*C_m_* (mg Cl/g paste)	13.52	25.68	20.72
	*C_s_* (mol/L)	3.60	3.67	3.52
	*B*	21.02	113.09	120.97
	Adjusted R-squared	0.995	0.995	0.998

**Table 5:** Thermogravimetry results of the paste samples.

Sample		Hydrate Content		AFm Compounds		Total Bound Chloride [mg/g Paste]	Bound Water [wt % of Sample]
		Ca(OH)_2_ Content [wt % of Sample]	AFm Content [wt % of Sample]	Interlayer Chloride Amount (*x*)	Chloride Content in AFm ^1^ [mg/g Paste]		
P	28 d	9.1	1.8–2.0	-	-	-	28.57
	0.05 M	8.6	-	-	-	3.8	26.00
	0.5 M	8.7	4.0–4.1	1.5	3.8	9.1	25.70
				2.0	5.1		
	3.0 M	8.2	8.6	2.0	8.6	62.2	25.05
A	28 d	~0.4	10.2–11.3	-	-	-	28.18
	0.05 M	~0.2	-	-	-	10.4	27.57
	0.5 M	~0	17.2–17.4	1.5	16.5	28.9	27.51
				2.0	22.0		
	3.0 M	~0	27.9	2.0	35.3	98.6	27.56
C	28 d	~0.4	4.4	-	-	-	28.81
	0.05 M	~0	-	-	-	9.3	28.61
	0.5 M	~0	8.5–8.6	1.5	8.1	22.0	27.02
				2.0	10.8		
	3.0 M	~0	20.4	2.0	25.8	93.3	26.36

^1^ The chloride content in the AFm compounds was calculated considering the mass fraction of the interlayer chemical composition [*x*Cl −·((2 − *x*)/*n*)other anion^n−^].

**Table 6:** Experimental results of the non-steady state chloride migration (NSSM) test.

Sample	Experimental Results (Migration Test)			*D_NSSM_* [×10^−12^ m^2^/s]		
	Initial Current [mA]	Applied Voltage [V]	Ingress Depth [mm]	Exp.	Average	Standard Deviation
PM-1	151	15	18.64	18.03	19.10	1.26
PM-2	142		20.41	20.49		
PM-3	142		18.92	18.77		
AM-1	268	10	10.09	13.47	14.41	0.90
AM-2	256		11.10	15.26		
AM-3	267		10.62	14.50		
CM-1	176	15	27.14	28.13	28.33	0.24
CM-2	165		27.50	28.25		
CM-3	164		27.78	28.60		

**Table 7:** Computed parameters of error function and half-Gaussian equation in the natural chloride diffusion test.

Sample	Error Function			Half-Gaussian Equation		
	*D_a_* [×10^−12^ m^2^/s]	*C_s_* [wt % of Dried mortar]	Adjusted R-Squared (Reduced Chi-Squared)	*D_i_* [×10^−12^ m^2^/s]	*M* [wt % of Dried Mortar]	Adjusted R-Squared (Reduced Chi-Squared)
PM	24.41	1.44	0.988 (0.73)	13.69	20.65	0.991 (0.89)
AM	15.14	2.94	0.929 (7.72)	11.35	35.18	0.940 (4.58)
CM	53.68	1.42	0.978 (0.26)	31.66	30.18	0.986 (0.21)

**Table 8:** Total amount of natural diffused chloride computed by the half-Gaussian diffusion model.

Sample	*m* [wt % of Dried Mortar]	*m_a_* [wt % of Dried Mortar]	Parameters of Shifted Half-Gaussian Equation				*m*/*m_a_*
			*a*	*b*	*D_is_·t*	*m_s_*	
PM	20.65	20.49	4.82 × 10^−3^	6.75	3.80× 10^−1^	1.01	1.01
AM	35.18	35.28	2.05 × 10^−5^	11.24	8.05× 10^−4^	0.09	1.00
CM	30.18	29.29	9.21 × 10^−3^	8.61	1.18	2.00	1.03

**Table 9:** Computed parameters of fitted curve using Holliday equation in the natural chloride diffusion test.

Sample	Weighted Regression Method	*R*_1_ [wt % of Dried Mortar]	*R*_3_ [mm]	Diffusion Coefficient [×10^−12^ m^2^/s]		Adjusted R-Squared	*m_h_*^1^/*m_a_*
				*D_h_*	Average (Except Non-Weight Method)		
PM	1/*y_i_*^2^	0.880	7.385	8.42	13.84 (11.04)	0.768	0.83
	1/*y_i_*	0.954	6.917	13.66		0.892	1.06
	non-weight	0.996	6.568	19.44		0.926	0.96
AM	1/*y_i_*^2^	0.425	9.685	3.84	9.28 (6.14)	0.600	1.12
	1/*y_i_*	0.508	10.312	8.43		0.800	1.03
	non-weight	0.559	10.870	15.56		0.854	1.04
CM	1/*y_i_*^2^	0.929	7.497	47.70	53.64 (50.73)	0.922	1.10
	1/*y_i_*	0.936	7.044	53.75		0.923	1.18
	non-weight	0.941	6.666	59.47		0.917	1.15

^1^*m_h_*: computed integral area using Holliday equation.
